# What If Your Husband Doesn’t Feel the Pressure? An Exploration of Women’s Involvement in WaSH Decision Making in Nyanchwa, Kenya

**DOI:** 10.3390/ijerph16101763

**Published:** 2019-05-18

**Authors:** Thelma Zulfawu Abu, Elijah Bisung, Susan J. Elliott

**Affiliations:** 1 Department of Geography and Environmental Management, University of Waterloo, 200 University Avenue West, Waterloo, ON N2L 3GI, Canada; susan.elliott@uwaterloo.ca; 2 School of Kinesiology and Health Studies, Queen’s University, 28 Division St, Kingston, ON K7L 3N6, Canada; eb120@queensu.ca

**Keywords:** women and girls, WaSH, participation, finance, Kenya

## Abstract

Access to water, sanitation and hygiene (WaSH) is a major challenge in sub-Saharan Africa (SSA). Women and girls suffer the main burden of a lack of access to WaSH because they are primarily responsible for collecting water for their homes. However, they are often excluded from WaSH decision-making and implementation processes. This research sought to explore women’s experiences in participating in WaSH decision-making through a case study in Nyanchwa, Kenya. Twelve (12) key informant interviews were conducted with community leaders and members regarding challenges and possible measures for enhancing women and girls’ participation in WaSH decision-making. From this research, it is evident that economic challenges and cultural factors such as male dominance, greatly inhibit women and girls’ participation in WaSH decision-making and implementation processes. Other factors such as time constraints and low literacy rates also emerged. The paper concludes with a call for collaboration among women’s groups to enhance collective action for improved access to WaSH. This will undoubtedly lead to enhanced community health and wellbeing (Sustainable Development Goal 3, SDG3) through the empowerment of women (Sustainable Development Goal 5, SDG5).

## 1. Introduction

Globally, 844 million people do not have access to a basic source of water [1]. In addition to inadequate access to safe water, it is estimated that 2.3 billion people do not have access to a basic sanitation service [1]. These challenges typically affect rural and marginalized residents, particularly in sub-Saharan Africa (SSA), where only 58 percent of rural populations practice good hygiene [1]. In addition, women and girls are disproportionately affected by inadequate access to safe water, sanitation and hygiene (WaSH). This is because they are often tasked with the responsibility of collecting water for their households and domestic chores, such as cleaning and cooking, that require the use of water. In sub-Saharan Africa, it is estimated that women and girls spend about 40 billion hours a year collecting water [2]. In most instances, this lost time could be directed towards economic activities or strengthening social ties [3]. Thus, during periods of water scarcity, women and girls struggle to fulfill their sociocultural obligations regarding house-keeping [4,5].

This challenge, and its impacts on the health and wellbeing of women and girls, gained global attention when it was included in the Sustainable Development Goals (SDGs). That is, SDG 6 target 6.2 aims to, “*achieve access to adequate and equitable sanitation and hygiene for all and end open defecation, paying special attention to the needs of women and girls and those in vulnerable situations*”. Similarly, SDG4 aims to, “*build and upgrade education facilities that are child, disability and gender sensitive and provide safe, non-violent, inclusive and effective learning environments for all*”. The role of access to WaSH in empowering women and ensuring gender equality has been well documented in recent studies [6,7,8]. For instance, in Nigeria, Jumare et al. [9] found that females, aged 16–20 years, reported late to school more often compared to boys, due to water collecting responsibilities at home. Similarly, Miiro et al. [10] observed a strong correlation between adequate menstrual hygiene infrastructure and school attendance in Uganda. In addition to water collecting responsibilities, adolescent girls also experienced psychosocial stressors, such as fear of being teased or of having stained uniforms, as reasons for staying away from school during menstruation. This implies that the lack of access to WaSH infrastructure in schools may result in educational inequalities as girls are more likely to drop out of school [11]. At the community level, Hirve et al. [12] looked at the psychological stressors associated with lack of access to sanitation in India. They reported that women who engaged in open defecation faced several psychosocial stresses including fear for their personal safety, injury or accidents, lack of cleanliness, indignity, and shame and embarrassment due to a lack of privacy [12]. Similarly, Sweetman and Medland [13], in their review of WaSH and gender in India, indicated that women and girls who engaged in open defecation are often abused and harassed by their male counterparts.

Despite girls and women bearing a disproportionate burden of inadequate access to WaSH, they are typically excluded from decision-making, implementation, and management of WaSH projects. For instance, Routray et al. [14] found that only 9% of sanitation decisions in India were made by women, and only 11% were made in consultation with women. The absence of women in the sanitation planning process was due to the low economic and social status of females within communities [14]. Women may lack the confidence to put forward ideas especially when they cannot finance them [14]. In situations where women are part of committees, their male counterparts often exclude them during meetings [15]. However, Hirai et al’s. [16] study on women’s involvement in WaSH decision-making in Kenya found positive links between women’s involvement and improved household sanitation uptake in households [16]. Similarly, in Senegal, the involvement of women in the management of the water supply networks has contributed to the significant increase in water coverage since 2004 [17]. The inclusion of women in water management teams in Senegal is strictly backed by government to ensure their concerns are included in planning and implementation [17].

**Figure 1:**
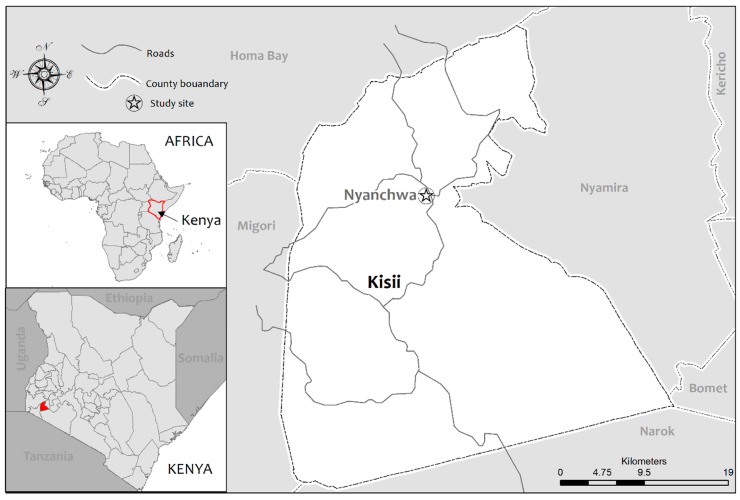
Study site: Kisii, Kenya.

There is currently limited literature focused on the economic and social power of women to take up roles in WaSH decision-making, particularly in the context of SSA. In light of this gap, this research aimed to understand women’s experiences in participating in decision-making around the implementation and management of WaSH infrastructure through a case study in Nyanchwa, Kenya (Figure 1). This paper is organized in seven sections. Subsequent to this introduction, the paper outlines the theoretical framework used to inform the research. This is followed by the study context and then the research design and methodology employed to address the research questions. The presentation of results is followed by a discussion of the links between the findings and related literature. The paper concludes with a call for collaboration among women’s groups to enhance collective action for improved access to WaSH. This will undoubtedly lead to enhanced community health and wellbeing (SDG3) through the empowerment of women (SDG5).

## 2. Theoretical Framework: Ecosocial Theory

Ecosocial theory seeks to explore the pattern of disease distribution and health by examining the combination of social processes and structures, cultural norms, ecological settings, and genetic variability and similarity among people in a region [18]. Within an ecosocial perspective, bodies incorporate and reflect social, ecological and biological processes such as structural and social inequalities [19]. These are reflected through embodiment which refers to the process through which humans literally incorporate, biologically, the material and social worlds in which they live [20]. Agency reflects the individual’s capacity to act to improve their own life and the need to take responsibility for any (in)actions. Finally, accountability directs attention to the (in)actions of stakeholders and the social systems that create inequalities such as lack of access to WaSH. This research sought to explore how lack of access to WaSH and barriers to women and girls’ participating in WaSH decision-making are biologically expressed in the Nyanchwa community. In addition, through the construct of agency, the study explored how the community, specifically women and girls, worked together to ensure access to safe WaSH. Others have employed ecosocial theory in a similar manner. For example, Krieger [21] in studying the effects of racial discrimination on the health outcomes of African American people in the United States of America, adopted the ecosocial theory. People of color biologically embodied social, biological and structural factors in their communities and this was expressed as poor access to quality healthcare. Bisung et al. [22] in a study in Usoma, Kenya, used ecosocial theory to examine how community members embodied unemployment, marginalization and unequal distribution of resources, which was expressed through a lack of access to WaSH in the community. The ecosocial framework was adapted by Kangmennaang and Elliott [23] in their research on understanding wellbeing in Ghana. From their study, it was evident that the definition of wellbeing differed by gender, ecological context and location across Ghana. Wellbeing was expressed as an embodiment of the social, economic, climatic and political structures. For example, some respondents described wellbeing as meeting their basic living standards without support from others, whilst others described it in terms of collective experiences and social support. In 2017, Baldwin et al. [24], through the lens of the ecosocial theory, established that the quality of healthcare experienced by sexual and gender minority women in the United States of America is characterized by external environmental factors such as nondiscrimination state legislation. In addition, sexual and gender minority women felt more comfortable disclosing their health needs to health workers in such states.

## 3. Study Context

This research was conducted in Nyanchwa, a rural community within the municipal county of Kisii, in Kenya, in SSA. Kenya is home to 46 million people, 58 percent of whom have access to a basic water source and 30 percent of whom rely on basic sanitation services [1]. The challenge of a lack of access to safe WaSH is more pronounced in the rural areas; the 2017 Joint Monitoring Programme (JMP) report indicated that 83 percent of urban households have access to basic water sources compared to only 50 percent of rural households. Due to the lack of WaSH, waterborne diseases such as diarrhea and cholera are prevalent. For instance, in 2017, cholera outbreaks affected about 2,800 people across six counties in Kenya [25].

In Kisii, the Gusii Water and Sanitation Company (GWASCO) is responsible for the provision of water and sanitation infrastructure and related services. However, only 22 percent of rural community members in Kenya are connected to the piped system and people are constantly faced with water rationing [1]. As a result, some community members use informal water vendors to meet their water supply needs. A number of communities in sub-Saharan Africa are affected by a lack of access to WaSH and the community selected, Nyanchwa, is just one of many. Most of the population acquires water from streams, springs and pipe-borne systems provided by vendors. The town is constantly affected by water scarcity due to unequal access to improved water sources. The situation is amplified by the fast-growing population and the contamination of surface water sources by activities such as car washing [26,27]. Thus, this community is experiencing a severe burden of lack of access to WaSH. In order to elicit a deep understanding of the factors contributing to women’s WaSH insecurities in SSA, it is essential that community-based work involve community partners who are fully engaged in the need for the research and can advocate for policy based on evidence produced. The research team has a strong relationship with a community partner that supported undertaking this research.

## 4. Methods

### 4.1. Data Collection

The data were collected in June 2016 using key informant (KI) interviews (*n* = 12) in the Nyanchwa community, with community leaders, members and various WaSH stakeholders. Key informants were recruited based on their knowledge, experiences, and engagement in the community’s activities. They were from institutions such as the community development committee, health center, primary school, high school, social development committee, and youth and women’s groups. Respondents were recruited using a snowball method. This involved community members directing the team to notable persons who had some knowledge on Nyanchwa community WaSH management. The researchers observed saturation after interviewing twelve (12) key informants from these various community groups. All interviews were conducted in English, except for one which was conducted in Gusii and later translated into English. Each interview lasted for about 90 minutes. All interviews were recorded with the permission of the interviewee and subsequently transcribed verbatim. Prior to data collection, the University of Waterloo Ethics Review Board approved the study (21484).

### 4.2. Data Analysis

The data were transcribed in English. Both descriptive and analytic coding were used to analyze the transcripts [28]. The coding was first done deductively, where themes were developed from the guiding theory as well as the emerging context, based on the goals of the study. Second, inductive coding was undertaken where themes emerged from the transcripts. The codes coalesced around three major themes: causes of lack of access to WaSH, the experiences of women and girls, and factors affecting the participation of women and girls in WaSH decision-making and management. The key themes were reviewed several times to ensure the themes and subthemes were coded in the same category. Portions of the transcript were coded on two different occasions to ensure consistency in the emerging themes. Results are organized around the three themes and the narratives are punctuated by direct quotes from the research participants.

## 5. Results

Participants represented a range of social backgrounds (Table 1). Table 2 presents themes that emerged from the interviews, the number of times they were mentioned and the number of respondents mentioning them. They were first categorized according to the causes of lack of WaSH in the community (economic challenges, leadership challenges, population growth and environmental challenges), the impacts of lack of WaSH on women and girls (time wasting, insecurity, disease, psychosocial impacts, and school dropout) and the factors that determined the participation of women in WaSH decision-making (male dominance, economic challenges, time constraints and a low literacy rate).

### 5.1. Causes of Lack of WaSH Infrastructure

#### 5.1.1. Economic Challenges

Access to water and sanitation services in Nyanchwa is not equal. The piped water system is connected to the village yet not all community members have piped water on their premises. The lack of access to water in general was associated with financial difficulties in purchasing equipment and paying for labor:

“*One is finance, because you cannot do anything without finances. I’m very sure that we are lacking finances. We cannot run down water by gravity unless we have the pipes for the connections and a pumping machine as well. So, we need money for all this and that is a major setback*”.

(Community member 1)

Furthermore, one major means of improving water access in homes is improving rainwater harvesting. It was clear that community members did not have the financial resources to engage in water storage:

“*Even those who have it (pipe borne water), it is not adequate and not regular. Some members of the community are poor and do not have money to buy a tank to store water*”.

(Community leader 5)

The story was no different for the sewerage connection system:

“*To connect to the sewerage line, there is a fee which you pay to the county government and then the laying of the pipes from your house to that place is also expensive*”.

(Community leader 8)

#### 5.1.2. Leadership Challenges

Access to water and sanitation is a right which should be provided by the state. However, some community members believed that leaders were not accountable and did not take responsibility toward ensuring access to water and sanitation:

“*I think it is lack of resources and our leaders being selfish*”.

(Community member 2)

Implementation of infrastructure such as WaSH is laden with issues of local politics and power:

“*I think one main challenge is the local politics and public politics. These politicians would always want to use the problem we mention for their political campaigns before election and they may derail projects*“.

(Community leader 5)

#### 5.1.3. Population Growth

The Nyanchwa community is experiencing a fast rate of population increase, yet development of infrastructure for the provision of safe WaSH to community members is progressing at a slow pace:

“*In the year 2012, when I came to Nyanchwa, the population was quite low. There has been development in this area, people have migrated to this place for development*”.

(Community leader 1)

The fast inflow of people into the Nyanchwa community has contributed to a severe lack of WaSH in the community:

“*The population of this place keeps on increasing. Nowadays we have a lot of people coming into the Nyanchwa community to live here which makes the water scare. Not enough for everyone*”.

(Community leader 7)

Also,

“*The sewerage system put in Nyanchwa area is actually a very old system that was meant for a smaller population. Maybe, that was for the sake of the small population at that time but now, the population is quite actually 10 times higher*”.

(Community leader 1)

#### 5.1.4. Environmental Challenges

Participants mentioned several forms of environmental challenges as causes of lack of access to WaSH. Nyanchwa has prolonged dry seasons and this affects the quantity and quality of water available to residents. The majority of the community relies on rainwater as it increases the level of the ground water table and increases the water quantity in the streams and rivers. During prolonged dry seasons, water is very scarce:

“*So, when people want to clean, they find that water is not sufficient for consumption and it is also not enough for cleaning of their homes, clothing and utensils. Others have gone as far as digging wells, but during the dry season, even those wells dry up*”.

(Community leader 2)

Similarly, the community relies on boreholes which are improved water sources as compared to open wells; however, during the dry season, these boreholes are not functional and are faced with similar challenges to those of the wells,

“*Boreholes too sometimes when there is a drought, the boreholes may become dry*”.

(Church leader)

### 5.2. Impacts of Lack of Access to WaSH on Women and Girls

#### 5.2.1. Wasted Time

Given that very few households were connected to the water system, most of the householders with no access to safe water in their homes needed to go in search of water and other sanitation services. Walking long distances to collect safe water is a necessary activity for women and girls in search of water. This process causes women to continually have less time for their businesses. Time- wasted was a major theme that emerged in this research:

“*So, this is a very big challenge for women and girls who walk to the river to go and fetch water. They actually spend most of their time looking for water. That means that in this community with the little supply of water women suffer most*”.

(Community leader 5)

Women and girls not only had to waste time walking long distances, but also had to wait in long queues especially at safe water sources such as boreholes and privatized piped water sources.

“*You would find our women and young children queuing in a line for water. It is time wasting for the women and the children*”.

(Community member 1)

The problem of time wasted for women and girls is crucial, as respondents continually emphasized how other duties were neglected to search for water:

“*Yes, they can come (to the water sources) at 7:00 am in the morning and they go back home at 2:00 pm*”.

(Community leader 7)

#### 5.2.2. Insecurity (Rape and Murder)

Women and girls are disproportionately faced with physical violence as a result of lack of access to safe WaSH. From the interviews, women expressed concerns with the distances traveled to access sanitation services. They also had concerns about the location of some of these WaSH facilities:

“*When they (women and girls) hide to go for defecation, the boys follow and rape them*”.

(Community Leader 3)

In addition, the lack of sanitation, and open defecation, were linked to other severe crimes perpetrated against women and girls, including murder:

“*There was a time a girl was murdered down there. She had gone for a call and did not come back home. Her body was found the next day. If we can have our toilet within the house, it will be good*”.

(Community Member 2)

In order to avoid long queues and water-related quarrels that occur at the point of collection, women sometimes woke up at odd hours to go and fetch water:

“*Even there are times when I see women being molested along the way. Because if you want to get to the water point as the first person, you have to wake up at midnight or at dawn. Not being in a group, this is how men molest women during those odd hours*”.

(Community member 3)

#### 5.2.3. Diseases

Community members mentioned WaSH-related diseases as key challenges they faced:

“*Without water you cannot wash your hands, therefore, as a result most of the children get diseases and most of the people in the community get diseases because of lack of water.*”

(Community leader 4)

Some of the girls acquired urinary tract infections and other related diseases due to the poor hygiene conditions of the WaSH facilities in their schools:

“*We came to realize that some girls are suffering because of our unhygienic conditions. Because it affects their private parts, they suffer and end up running away from the school and some of them may look for a school that is probably better than ours*”.

(Community leader 5)

#### 5.2.4. Psychosocial Impacts

Aside from physical abuse and harassment, women expressed a lot of stresses in searching for safe WaSH. The burden of water collection coupled with other household responsibilities put pressure on women:

“*These mothers are the same mothers who have been cooking for us. These mothers are the same mothers who are washing our clothes and everything else. When we have less water within, we put more pressure on them to do more with less*”.

(Community leader 1)

Another community leader added:

“*For them (women) they need a lot of water and probably all the time. So, I believe for them it weighs on them more than it does for men*”.

(Community leader 8)

Women require safe WaSH to keep the home hygienic. Women also need water for their hygienic purposes, especially during their menstrual periods. However, the absence of water to keep their bodies clean made them worry that they might smell in public. The lack of WaSH also affected the duration of time spent on generating income:

“*So, there is that fear of moving about and one becomes restless because you need to go out and make money. Which you cannot do because of the fear that you might be smelling bad without knowing. In fact, going to school (her work place) sometimes is a problem on its own*”.

(Community member 3)

#### 5.2.5. School Drop-Out

Given the cultural duty of the women to provide water, many of them prioritize this responsibility to the detriment of other important activities. For example, given that water sources are less crowded by midday, girls took advantage of this to fetch water at the expense of their education:

“*In our culture, water should be brought home by the females who are the mothers and the young girls. You would find kids leaving school to go and fetch water especially during the dry season*”.

(Community leader 8)

There were concerns about some students who dropped out of school entirely because they missed a lot of school hours due to their responsibility for collecting water:

“*So, these girls have to waste much of their time looking for water and then now they go to school late. So you get most of the girls dropping out of school because of going to look for water and then now dropping out of school becomes also a problem…Most of the girls (in the community) are not educated*”.

(Community leader 4)

Furthermore, girls quit or dropped out of school when they reached the age of menstruation. Girls at this age are very shy and concerned about their appearance. The absence of, or limited access to, menstrual changing facilities or toilets prevented some girls from coming to school. At this time, girls avoided school for fear of staining their clothing.

### 5.3. Barriers to Women’s Participation in Improving WaSH

Women and girls have key roles to play with regards to improving access to WaSH. Women constantly engage in collecting water and hence have knowledge regarding access, and the quality and quantity of water. However, women are typically absent in decision-making processes in many communities. When asked about barriers to women’s participation in water-related decision-making, respondents primarily mentioned male dominance, economic challenges, time constraints and low literacy rates among women.

#### 5.3.1. Male Dominance

Culture plays a significant role in the kind of activities women participate in. According to respondents, women often had to seek permission from their husbands to be able to participate actively in some occasions. In this context, the man is the head of the household:

“*Yes. Naturally in Kisii land, a woman is under a man. She is under a man and if they are not permitted to go, she can’t go. Even when we have been having this community work, maybe repairing a well, mostly we call the women to come and fetch the stones. If they are not permitted, then they wouldn’t come. This is a major problem because they are not alone, they must seek permission and when it has been granted, then they can go*”.

(Community member 1)

Women and girls not only needed the permission of their husbands and fathers to participate but also to join some social groups that might help solve their needs:

“*The challenge is that even if these women have the idea, but would their husbands permit them to join such groups [social groups]?*”

(Community member 3)

Similarly,

“*When we try to make decisions, they always say let us consult our husbands because they have the final say in the house*”.

(Church leader)

The males were most often left to talk on behalf of the women, which was likely not a true reflection of the issue:

“*What if your husband does not feel the pressure that you feel?… So especially our women many at times they put these men to decide. They are not able to participate actively and even when they sit with their husbands in the same meeting, they expect the husband to talk about water and not them (women) as a person who is already in this (water challenges)*”.

(Community leader 1)

#### 5.3.2. Economic Challenges

Women continue to refrain from decision-making gatherings because they may not have the resources to put their decisions into action:

“*As I have experienced in the past, they (women) say we want ABCD with regards to the availability of water, but when it comes to money, they direct you to their husbands*”.

(Community leader 3)

In some cases, community members are expected to make contributions for the construction of boreholes and other WaSH infrastructure. In most cases, women avoided such gatherings since they did not have the financial independence to make these commitments. For instance, a community member said:

“*Even if we talk of the financial contributions, they may shy off because of their low financial bargain*”.

(Community leader 1)

Due to the fact that women could not contribute financially to the implementation of projects, their role was mainly limited to manual labor:

“*In most cases, you find that they (women) are providing labor. For instance, our spring was bushy, muddy and full of dry leaves around it. I had to make them (community members) clean and the majority that I had there were women. They were the ones I used in cleaning the place. So that is the place they can do a lot better because when it comes to finance, it goes to their husbands or their fathers*”.

(Community leader 3)

#### 5.3.3. Time Constraints

As culture demands, women play very important roles in organizing domestic activities. They are responsible for the upkeep of their families, hindering their participation in community activities:

“*There are challenges because as I told you, much of the work in our homes according to our culture is done by the women. So, as they use much of their time looking for water, it becomes impossible for them to attend “Barazas*” (community meetings)”.

(Community leader 4)

Women who worked for themselves were mostly engaged in hawking (selling at street stalls) to help get some money to sustain themselves, hence they may not have had the time to participate:

“*Most of the women and young ladies are involved in business to get some little money. So, by the time they come back to their houses, it is late in the evening*”.

(Community member 2)

Also, women were engaged in multiple activities at the same time:

“*The woman may be a teacher at the same time babysitting her baby and keeping the household. The challenges of time may also hinder her from participating*”.

(Community member 3)

#### 5.3.4. Low Literacy Rates

Even though women are recognized as very important in decision-making, one of the respondents attributed the fact that women did not participate in WaSH gatherings to the mode of communication used:

“*Lack of education also becomes a problem. Because if one is not educated, he or she sees herself as inferior to join others in public barazas. Because in public barazas we use two languages—Swahili and English. So, if this girl or mother is not literate sometimes, they stay away because of the language that is being used*”.

(Community leader 4)

Another respondent indicated that literacy played a major role in these decision-making gatherings:

“*I think maybe lack of knowledge. Most of them their low level of education prevents them from expressing themselves clearly. Some may be shy because in the community, men are the ones who are in front to even solve the problems of women*”.

(Community leader 5)

## 6. Discussion 

The importance of women’s role in WaSH service provision cannot be over emphasized. Over the years, several challenges (economic challenges, climate change, poor leadership and population increase) have increased the burden of lack of access to water, especially in the dry season. These challenges are a combination of various broader structural and environmental challenges.

From this research, guided by the ecosocial theory, it was evident that the lack of safe WaSH, coupled with the lack of women’s participation in WaSH decision-making, is expressed in negative outcomes such as insecurity, higher rates of school dropout, disease acquisition, psychosocial impacts and wasted time among women and girls. As these results show, the scarcity of water and sanitation services has several impacts on girls and women, both in school and at home. For instance, women could be engaged in other economically and socially valuable activities as opposed to searching for water. Furthermore, it was evident that the few women who were engaged in some economic activities were losing out economically because the most productive time of the day was wasted in searching for water. The situation was no different for girls attending schools in the Nyanchwa community. The lack of WaSH at the community level, and the lack of WaSH in the school environment, were critical factors in girls not staying in school. Firstly, at the community level, especially during dry seasons, girls left school during the day in search for water for their homes. At these times, the water sources were less crowded as many women returned home to engage in other domestic activities. Secondly, girls in situations where lack of WaSH was a major problem were not free from psychosocial stressors. In this research, girls were compelled to leave school to fulfil their domestic duty of ensuring adequate access to water in their homes. Further, the availability of water in their homes made it easier to perform other domestic roles, such as cleaning. Some girls did not return to school for fear of being punished. Similar to previous studies in India, it was observed that many young girls dropped out of school because of the lack of access to WaSH in this community. For example, Kookana et al. [29] found that more than 50 percent of girls were engaged in household chores and either reported to school late or missed out on school altogether.

The lack of access to WaSH services in some schools, as previously mentioned, contributed significantly to girls dropping out of school. In this study, the community schools lacked appropriate WaSH facilities, hence girls who were menstruating missed school days because they would rather stay at home where they could manage their period and keep themselves clean. On the contrary, Rajagopal and Mathur [30] in their study on the experiences and challenges faced by adolescent girls in managing menstruation in school and at home in slums in India, found that 83.8 percent of girls did not miss out on school during menstruation. However, they faced a greater challenge with unhygienic toilets due to a lack of running water and inadequate waste management services. Unhygenic sanitation infrastructure is a major challenge in the Nyanchwa community. Schoolgirls develop diseases such as urinary tract infections from using unhygenic sanitary infrastructure in schools. The burden of overcoming these diseases contributes to girls dropping out of school. Furthermore, psychosocial impacts such as shyness, or fear of a stained uniform or body odor are struggles for many young females. These stressors have been identified at the community level and are detrimental to health and wellbeing [31]. Morgan et al. [32], in assessing the low coverage of WaSH in schools across six sub-Saharan countries including Kenya, indicated that there is low access to WaSH, especially menstrual hygiene services and low student-to-latrine ratios for girls as compared to boys. Cairns et al. [7] have indicated that lack of access to WaSH is critical to girls’ enrolment and school attendance. This is a major challenge hindering girls from attaining their full potential. Access to improved change rooms and self-care is very important in boosting girls’ confidence and granting them the peace of mind to study and contribute confidently in class.

This impact of high rates of female school dropout as a result of lack of access to safe WaSH is directly linked to the low rate of female literacy, especially in SSA. According to UNESCO, only 2/3 of people in the world are said to be literate. The regional Gender Parity Index (GPI) for sub-Saharan Africa is 0.92, indicating that there are more literate men compared to women. From this study, this further impacted the participation of women and girls in WaSH decision-making

When safe sanitation services are lacking, women search for safe hiding spaces to engage in sanitation and hygiene practices. Sommer et al. [33] indicated that open-air sanitary facilities, insufficient bathing areas, and lack of lighting at night intensified the risks of sexual violence for those using unimproved facilities. Women also risk losing their lives as reported in this research. Regardless of all these hazards, females in marginalized areas continue to engage in open defecation due to limited sanitation facilities [32]. Similarly, Khanna and Das [34] in their study in India, reported that the poorly designed and constructed nature of WaSH facilities which lacked safety—e.g., lacking seats or slabs, poorly lit or without locks—were the reason women did not use the sanitation facilities provided but engaged in open defecation. In this study, as a measure to ensure security, women accessed WaSH services in groups especially during the nighttime hours.

Despite the many challenges faced by women without adequate WaSH access, they are rarely involved in making decisions around WaSH. In this study, the barriers to participating in WaSH decision-making were interlinked and reinforced each other. For example, barriers such as male dominance and the economic stability of women are key to the way roles of women or girls are shaped. Men are typically the heads of their households and are seen as providers for their homes. To engage in activities, many women must seek the permission of their husbands and fathers. The trend persists because women are not financially independent. Not being financially independent limits women’s contribution to specific types of WaSH infrastructure that meets their needs. Access to WaSH is very important for women’s empowerment since they are the key players in water collection and hygiene improvement. Linked to the economic challenge are time constraints. In this research, some women complained of having less time available to engage in economic activities to generate their own income as a result of the lack of WaSH, and the need to perform other domestic activities. However, some studies have shown that women do not necessarily take up economically profitable jobs even though the hours spent on water collection may reduce [35]. Koolwal and Walle [36] in their research indicated that there was no evidence that improved access to water leads to greater off-farm work for women. From this research it was also evident that some women did not engage in WaSH decision making due to their economic activities. It was interesting to find out that some women did not have the time because they engaged in hawking (selling at street stalls). These women preferred to generate money than to attend WaSH meetings. Other women were engaged in household duties such as cooking and cleaning hence did not make it to such meetings. Improving access to WaSH has a great impact in a community but to what extent does that empower women? From this research it is evident that women need to engage in some economic activities since financial independence is a major determinant of wellbeing. Also, it is important to comprehensively empower women economically and socially to help address the power imbalance in participation around WaSH decision-making. For instance, Leder et al. [37] assessed two internationally funded programs aimed at empowering women by improving access to water for both domestic and productive use in Nepal. The projects failed to acknowledge and address how the dynamic interplay of cultural norms, age, marital status, gender roles and power relations shaped access to, and control over, water resources. Some women did not attend training sessions because they did not have the permission of their mothers-in-law or husbands. The two projects failed to achieve their purpose.

From this research, some impacts of the lack of WaSH translated into barriers to women’s participation in WaSH provision in the community. As previously discussed, girls who lack access to adequate WaSH are dropping out of school. A future effect of this is the high rate of illiteracy among women. Women and girls are barely seen at decision-making gatherings, and even when present seldom contribute because they cannot fully express themselves in English or Swahili, which are the main languages used during community meetings in Nyanchwa. This makes it hard to follow the conversation when participating in such meetings, and acquiring a translator to help share one’s thoughts can be burdensome. This makes a lot of the women feel shy and inferior.

From a policy perspective, there is a need for a deeper understanding of participation of women and girls in WaSH decision-making, and of social and economic empowerment. Women and girls need to be in control, and to participate in the management and provision of WaSH services in their communities. Governments and other relevant WaSH stakeholders in SSA such as international non-governmental organizations need to develop strategies that seek to address women’s economic and social needs alongside ensuring access to WaSH. These policies and programs must be informed by context and comprehensive research guided by social theory to be effective. Research has indicated that SSA and other developing regions have had several interventions including WaSH programs aimed at changing behaviors and improving health and wellbeing. These interventions are often not sustainable and rarely result in any real change [37,38]. Improving access to WaSH and empowering communities, especially women, to be able to sustainably manage WaSH infrastructure will ensure its longevity. Furthermore, it is critical to empower women economically in ways that permit them to significantly contribute to WaSH decision-making and implementation in their communities. It is critical for policies on WaSH and gender to include women’s empowerment through social groups. Research has shown that building social networks and trust among community members improves WaSH access and management [22,39]. In this research, not all women belonged to these social groups because they were not permitted by their husbands. However, these groups have the potential to give women financial power and a unified voice to participate in WaSH decision-making and implementation processes. Hence, policies and programs should also target other environmental issues, such as family hierarchy, to ensure the importance of WaSH and the involvement of women are clearly understood and more widely accepted.

## 7. Conclusions

From this research, it can be concluded that there is a major deficit of WaSH in the Nyanchwa community. Lack of access to WaSH can be attributed to economic challenges, leadership challenges, population growth and environmental circumstances. Women and girls are the most affected since they are required to collect water for their homes and to keep their homes clean. The greatest impact borne by women and girls was wasted time. However, women were often excluded from the WaSH decision-making and implementation process in the Nyanchwa community. In this research, the greatest barriers to participating in WaSH decision-making were male dominance and economic challenges. These barriers are interrelated and perpetuate a cycle of dependency and disproportionate exposure of women and girls to water-related insecurities. Low literacy rates as a barrier to participating in WaSH meetings among women is a consequence of girls dropping out of school. Also, time wasted in searching for safe water affects women’s ability to be economically productive. Finally, some women did not attend WaSH meetings as they were engaged in economic activities. This study demonstrates, the importance of having policies that holistically empower women (SDG5) whilst ensuring access to safe WaSH in home and schools (SDG6) to aid women and girls to fully achieve their potential. Together these will significantly enhance community health and wellbeing (SDG3).

## Figures and Tables

**Table 1:** Respondent characteristics.

Participants Pseudonyms	Length of Stay in Years in Community	Organization	
Marcus	5	Health care	Community leader
Matilda	68	Women’s group	Community leader
Adams	65	Education	Community leader
Julius	5	Business	Community leader
Osman	19	Public sector	Church leader
Joseph	8	Education	Community member
Ben	20	Religious group	Community leader
Noella	14	Education	Community member
Yussif	6	Education	Community leader
Rachael	5	Education	Community member
Abraham	9	Car washer/ water vendor	Community leader
Robert	3	Education	Community leader

**Table 2:** Themes emerging around water, sanitation and hygiene (WaSH).

Response	Number of Mentions (Number of Participants)
Causes of lack of improved WaSH	
Economic challenges	55 (12)
Leadership challenges	33 (9)
Population growth	28 (12)
Environmental circumstances	27 (11)
Impacts of lack of access to WaSH	
Time wasting	38 (9)
Insecurity	34 (7)
Disease	24 (7)
Psychosocial Impacts	22 (8)
School dropout	15 (6)
Challenges surrounding women’s participation in WaSH decision making	
Male dominance	22 (8)
Economic challenges	18 (7)
Time constraints	11 (6)
Low literacy rate	9 (4)
